# Dermal bacterial LPS-stimulation reduces susceptibility to intradermal *Trypanosoma brucei* infection

**DOI:** 10.1038/s41598-021-89053-2

**Published:** 2021-05-10

**Authors:** Omar A. Alfituri, Enock M. Mararo, Pieter C. Steketee, Liam J. Morrison, Neil A. Mabbott

**Affiliations:** The Roslin Institute and Royal (Dick) School of Veterinary Sciences, University of Edinburgh, Edinburgh, UK

**Keywords:** Immunology, Infectious diseases, Parasitic infection

## Abstract

Infections with *Trypanosoma brucei* sp. are established after the injection of metacyclic trypomastigotes into the skin dermis by the tsetse fly vector. The parasites then gain access to the local lymphatic vessels to infect the local draining lymph nodes and disseminate systemically via the bloodstream. Macrophages are considered to play an important role in host protection during the early stage of systemic trypanosome infections. Macrophages are abundant in the skin dermis, but relatively little is known of their impact on susceptibility to intradermal (ID) trypanosome infections. We show that although dermal injection of colony stimulating factor 1 (CSF1) increased the local abundance of macrophages in the skin, this did not affect susceptibility to ID *T. brucei* infection. However, bacterial LPS-stimulation in the dermis prior to ID trypanosome infection significantly reduced disease susceptibility. In vitro assays showed that LPS-stimulated macrophage-like RAW264.7 cells had enhanced cytotoxicity towards *T. brucei*, implying that dermal LPS-treatment may similarly enhance the ability of dermal macrophages to eliminate ID injected *T. brucei* parasites in the skin. A thorough understanding of the factors that reduce susceptibility to ID injected *T. brucei* infections may lead to the development of novel strategies to help reduce the transmission of African trypanosomes.

## Introduction

African trypanosomes are single-celled protozoan parasites that are transmitted between mammals throughout sub-Saharan Africa by blood-feeding tsetse flies of the genus *Glossina*. Human African trypanosomiasis is caused by infection with the *Trypanosoma brucei rhodesiense* and *T. b. gambiense* sub-species, whereas animal African trypanosomiasis is caused by infection with *T. congolense*, *T. vivax* and *T. b. brucei*. The trypanosome life-cycle within the mammalian host begins after the intradermal (ID) injection of metacyclic trypomastigote forms into the skin dermis by blood-feeding tsetse flies. Once within the skin dermis, the parasites undergo adaptation to the mammalian host and directly invade the lymphatics^1,2^ to access the local draining lymph nodes, before disseminating systemically via the bloodstream. During this initial establishment phase, the parasites also undergo a morphological change into the long-slender blood-stream forms that exist entirely extracellularly within the mammalian host^2–5^.

Much of our understanding of the host–pathogen interactions that influence mammalian trypanosome infections has been obtained from the study of experimental transmissions to mice infected via the intraperitoneal or intravenous routes. However, relatively little is known of the factors that are important in controlling disease progression following the natural ID route of infection. Mononuclear phagocytes (MNP) are a heterogeneous population of monocytes, macrophages, classical dendritic cells and tissue-specific phagocytes including the Kupffer cells in the liver, the microglia in the brain and the Langerhans cells of the skin epidermis. These cells are derived from haematopoietic precursors and are dependent on stimulation from the cytokine colony stimulating factor 1 (CSF1) for their development^6^. Kupffer cells in mice can readily phagocytose antibody-opsonized *T. congolense* parasites^7^, and data from experimental systemic transmissions show that macrophages play an important role in protection during the early stage of trypanosome infections by helping to limit the magnitude of the first parasitaemic wave^8,9^. MNP are abundant in the skin dermis, but little is known of their impact on ID trypanosome infections. The gradual accumulation of monocytes in the dermis after ID trypanosome injection has been reported, but these cells were not considered to play a significant role in parasite clearance^10^. Therefore, in the current study we determined the separate effects of increased abundance of MNP in the dermis and dermal LPS-stimulation on susceptibility to ID infection with *T. brucei* parasites in mice.

## Results

### Intradermal CSF1-Fc treatment increases the local abundance of CSF1R + MNP in the skin

**Figure 1:**
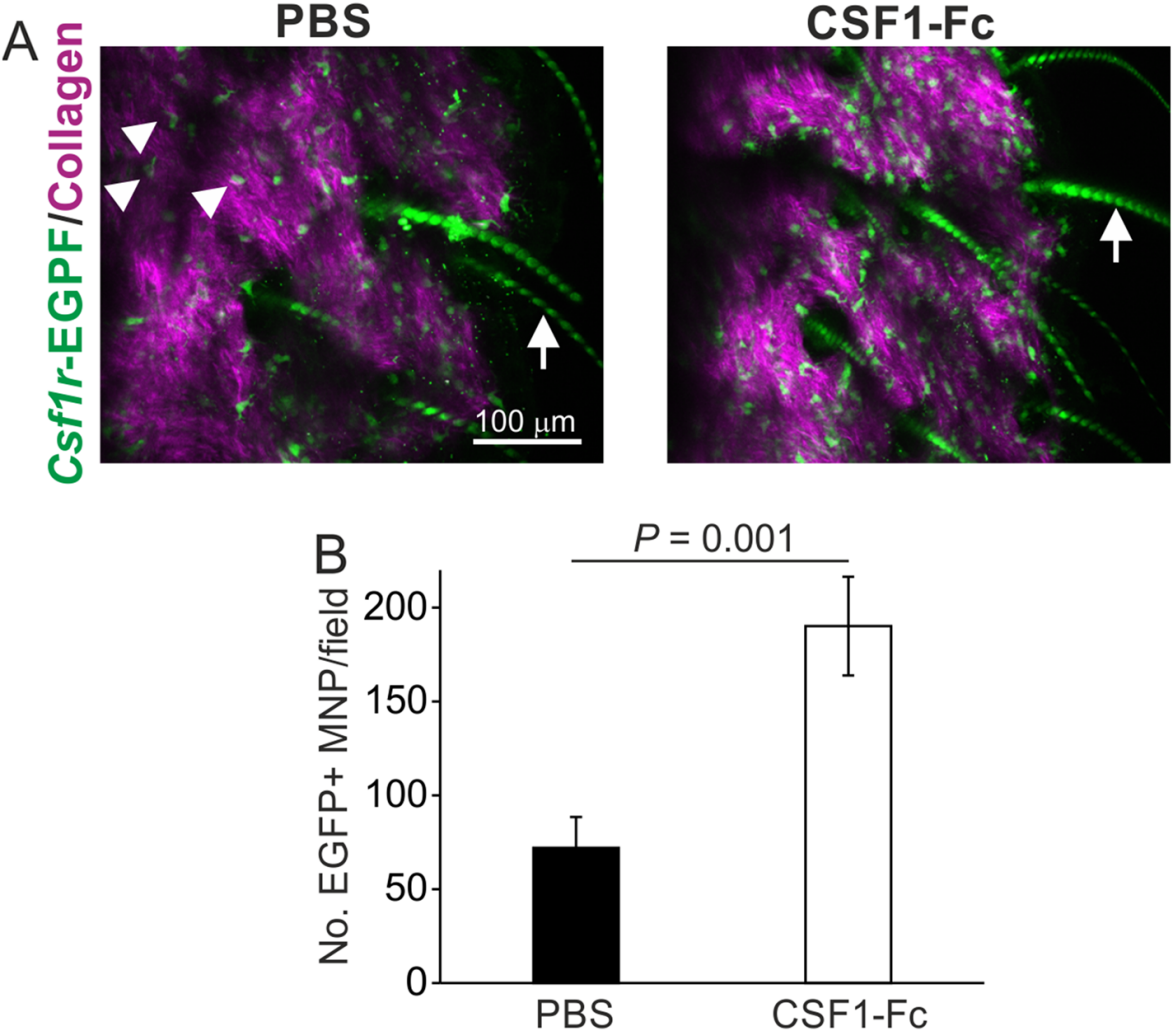
Enhanced abundance of Csf1r-EGFP + mononuclear phagocytes (MNP) in the dermis of Csf1r-EGFP^+^ mice after intradermal (ID) injection with CSF1-Fc. (**A**) Csf1r-EGFP reporter mice were injected ID with CSF1-Fc, or PBS as a control, for three days and 24 h after the final injection the ears were removed and viewed using a 2-photon microscope. Images show EGFP + MNP (green, arrowheads) and background collagen signal (purple) in the dermis. Arrows, auto-fluorescent hair follicles. (**B**) The numbers of EGFP + MNP in the dermis local to the injection site were significantly increased after CSF1-Fc-treatment when compared to PBS-treated controls (P = 0.001, Student’s t-test; CSF1-Fc, n = 4; PBS, n = 3).

Systemic administration of a CSF1-Fc conjugate significantly expands the abundance of colony stimulating factor 1 receptor (CSF1R)-expressing MNP in tissues^11^. We therefore used transgenic *Csf1r*-EGFP reporter mice to determine the effects of ID CSF1-Fc treatment on the local abundance of CSF1R + MNP in the skin. In these mice, EGFP expression is driven by the *Csf1r* promoter and stably and reliably reflects the expression of CSF1R protein^12^. In tissues from *Csf1r*-EGFP reporter mice all the MNP populations are labelled, even under circumstances where *Csf1r* mRNA is depleted^12^. The only other EGFP-expressing cells in these mice are the trophoblasts in the placenta that express *Csf1r* from a unique promoter. To restrict the effects of CSF1-administration to the dermis, groups of four mice were injected ID into the ear pinna with CSF1-Fc, or an equivalent volume of PBS as a control, for 3 days. Approximately 24 h after the final CSF1-Fc treatment the abundance of *Csf1r*-EGFP^+^ MNP in the vicinity of the injection site was then quantified microscopically (Fig. 1A). This analysis showed that the number of EGFP^+^ MNP in the dermis was significantly increased in CSF1-Fc-treated *Csf1r*-EGFP^+^ mice when compared to PBS-treated controls (Fig. 1B; *P* = 0.001, Student’s t-test).

### Intradermal CSF1-Fc treatment does not affect susceptibility to ID *T. brucei* infection

**Figure 2:**
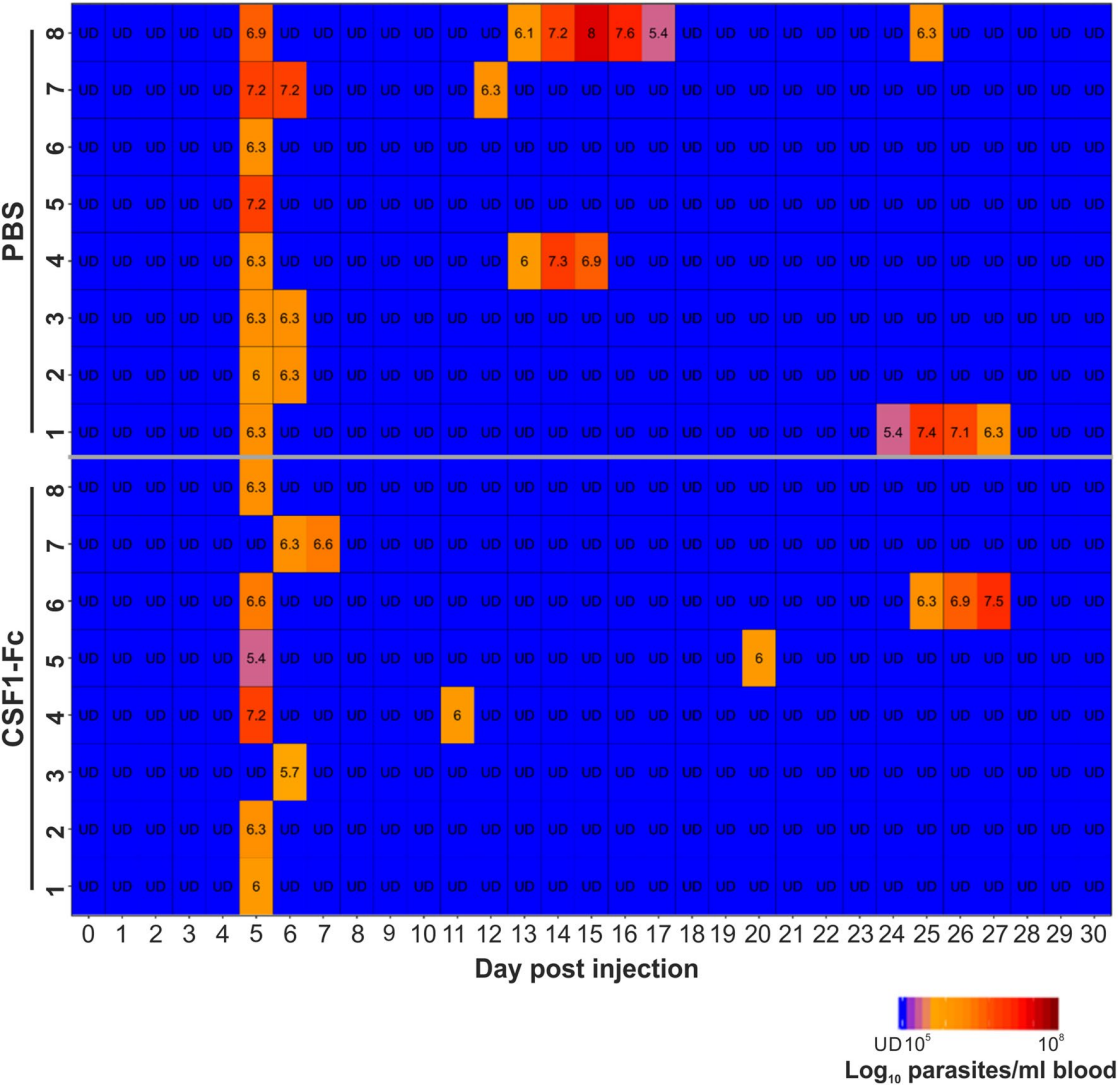
Intradermal CSF1-Fc treatment does not affect susceptibility to ID *T. brucei* infection. Groups of C57BL/6J mice were injected ID with CSF1-Fc, or PBS as a control, for three consecutive days and 24 h after the final injection the mice were injected ID with a 1 × 10^5^ dose of *T. brucei* STIB 247 parasites (*n* = 8 mice/group). Heatmap shows the log_10_ number of trypanosomes/mL of blood on each day after ID infection. Each row represents an individual mouse. UD = below detection limit (~ 5.4 log_10_ parasites/mL blood).

Next, groups of eight non-transgenic C57BL/6J mice were injected ID into the ear pinna with CSF1-Fc, or PBS as a control, as above, and 24 h after the final CSF1-Fc treatment the mice were injected ID with 1 × 10^5^ T*. brucei* STIB 247 parasites into the same site. Blood parasitaemias were then measured at daily intervals for 30 d post-infection using the rapid matching method^13^, which has a minimum detection limit of approximately 4 × 10^5^ parasites/mL of blood. These data showed that parasite kinetics after ID trypanosome injection were similar in mice from each treatment group. All the CSF1-Fc-treated and PBS-treated mice developed a parasitaemia that was first detectable from around 5 d post-infection, with similar parasite burdens detected at the peak of the first parasitaemia wave (Fig. 2; PBS 6 × 10^6^ parasites/mL; CSF1-Fc, 3 × 10^6^ parasites/mL; *P* = 0.1984, Student’s t-test, *n* = 8). Following the initial parasitaemia wave, a similar proportion of the mice in each treatment group displayed transient relapses in their parasitaemias during the remainder of the experiment (PBS, 4/8 mice; CSF1-Fc, 3/8 mice). Together, these data show that although the local abundance of CSF1R + MNP increased significantly in the dermis after CSF1-Fc treatment, this did not affect susceptibility to ID *T. brucei* infection.

### ID LPS treatment decreases susceptibility to ID *T. brucei* infection

**Figure 3:**
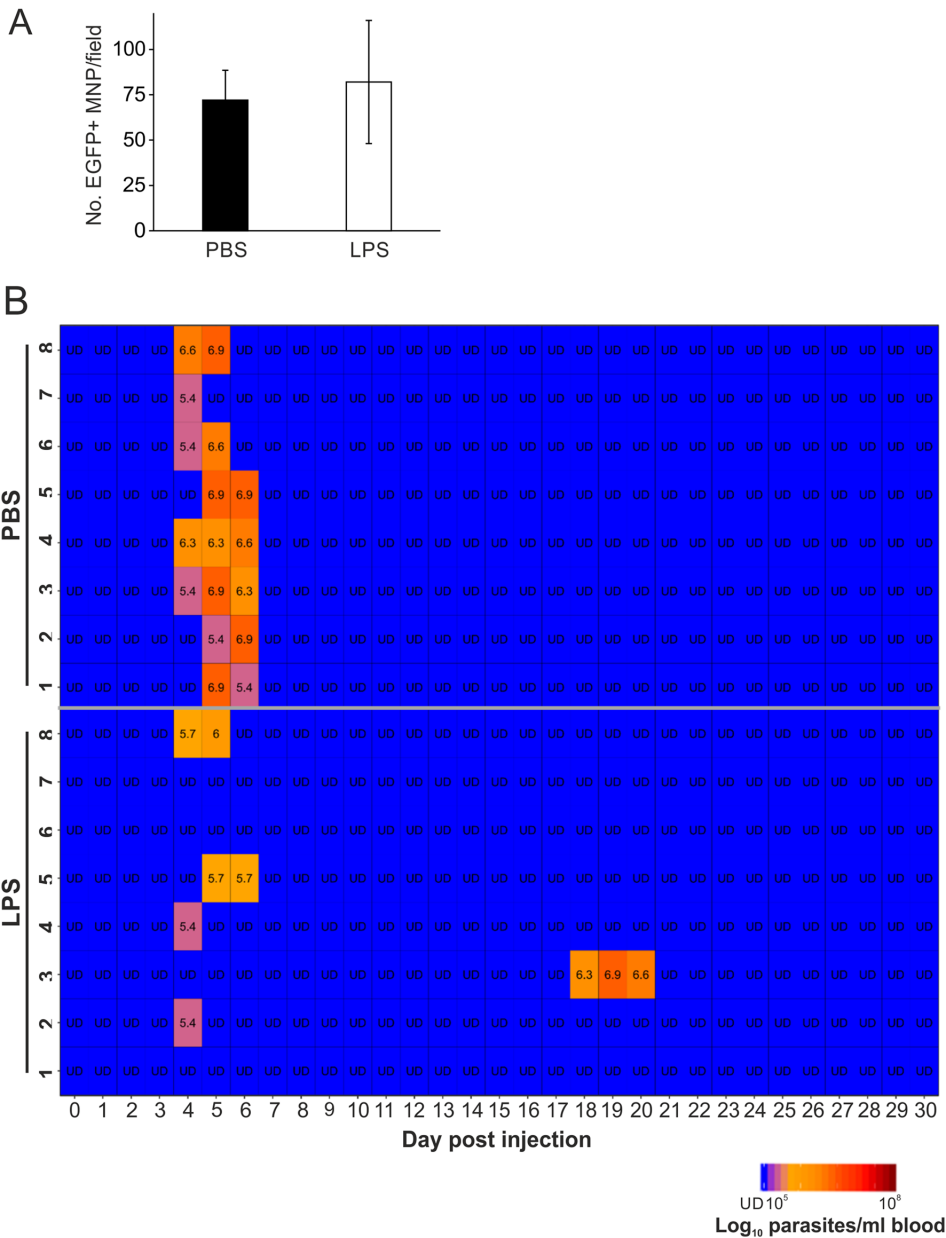
ID LPS treatment decreases susceptibility to ID *T. brucei* infection. (**A**) *Csf1r*-EGFP reporter mice were injected ID with LPS, or PBS as a control, and 24 h later the abundance of EGFP + MNP cells in the dermis local to the injection site determined as in Fig. 1. Similar numbers of EGFP^+^ MNP were observed in the ear following ID LPS or PBS treatment (*n* = 2–3 mice/group). (**B**) Groups of C57BL/6 J mice were injected ID with LPS, or PBS as a control, and 24 h later injected with ID with a 1 × 10^5^ dose of *T. brucei* STIB 247 parasites (*n* = 8 mice/group). Heatmap shows the log_10_ number of trypanosomes/mL of blood on each day after ID infection. Each row represents an individual mouse. UD = below detection limit of 5.4 log_10_ parasites/mL.

We next determined whether LPS-stimulation directly within the dermis would influence susceptibility to ID *T. brucei* infection. Groups of *Csf1r*-EGFP reporter mice were injected ID with LPS, or an equivalent volume of PBS as a control, and 24 h later the abundance of *Csf1r*-EGFP + MNP cells in the dermis local to the injection site was determined as above. This analysis suggested that ID LPS exposure did not affect the local abundance of *Csf1r*-EGFP^+^ MNP as similar numbers were observed in the dermis of PBS-treated control mice (Fig. 3A).

To determine the effects of ID LPS-stimulation on susceptibility to ID trypanosome infection, groups of C57BL/6J mice were first injected ID into the ear pinna with LPS (or PBS as a control) and 24 h later injected ID with 1 × 10^5^ T*. brucei* STIB 247 parasites into the same site. The blood parasitaemia was then monitored for 30 d. Whereas all of the PBS-treated mice developed a detectable parasitaemia, only 5/8 LPS-treated mice had detectable parasitaemias during the 30 d observation period (Fig. 3B). The magnitude of the mean parasite burdens at the peak of the first wave in those mice that developed a detectable parasitaemia also differed significantly between treatment groups (PBS, 6 × 10^6^ parasites/mL; LPS, 8 × 10^5^ parasites/mL; *P* = 0.0365, Student’s t-test). These data demonstrate that LPS-stimulation directly with the skin dermis significantly reduces susceptibility to ID trypanosome infection.

### LPS treatment enhances the ability of macrophage-like RAW264.7 cells to kill *T. brucei *in vitro

**Figure 4:**
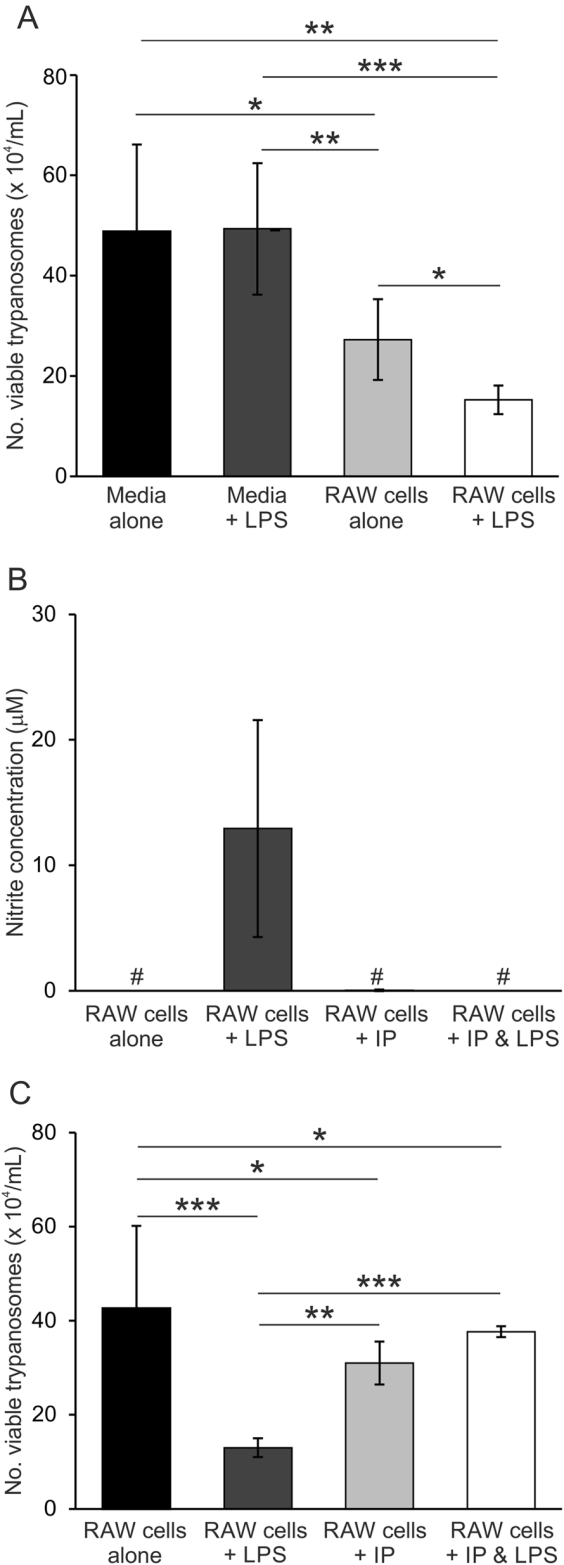
Enhanced ability of LPS-stimulated RAW264.7 cells to kill *T. brucei*. (**A**) Macrophage-like RAW264.7 cells were stimulated with 10 µg LPS (or PBS as a control), co-cultivated with *T. brucei* STIB 247 parasites, and the number of viable parasites determined 24 h later. (**B**,**C**) Macrophage-like RAW264.7 cells were stimulated with 10 µg LPS and/or 1 mM indolepyruvate, and then co-cultivated with *T. brucei* STIB 247 parasites. Twenty four hours later (**B**) nitrite levels in the culture supernatant, and (**C**) the number of viable parasites were determined. Data are shown as the mean ± SD for 3 wells/group from an individual experiment, and are representative of data from 3 independent experiments. *, *P* < 0.05; **, *P* < 0.01; ***, *P* < 0.001; # below the limit of detection.

MNP have been suggested to play an important role in trypanosome infections by phagocytosing and destroying the parasites and via the production of cytotoxic/cytostatic mediators^14,15^. Bacterial LPS is a potent pathogen-associated molecular pattern that elicits a strong immune response in MNP via Toll-like receptor (TLR)-4 signalling^16^. Thus, although LPS treatment did not affect the abundance of CSF1R + MNP in the dermis, it is plausible that the pro-inflammatory properties of LPS-stimulated MNP enhanced their ability to clear the parasites from the injection site and thus reduce the initial parasitaemic peak. To test this hypothesis, murine macrophage-like RAW264.7 cells^17^ were stimulated with LPS (or PBS as a control) and co-cultivated in vitro with *T. brucei* STIB 247 parasites. The number of viable parasites was then determined 24 h later. Stimulation with LPS enhanced the ability of RAW264.7 cells to kill *T. brucei* as the number of viable trypanosomes detected after co-cultivation in the presence of these cells was significantly reduced (Fig. 4A).

Trypanosome-derived ketoacids such as indolepyruvate can reduce the production of inflammatory mediators by LPS-stimulated macrophages including pro-inflammatory cytokines and the toxic free radical nitric oxide (NO)^18,19^. Production and excretion of these metabolites may enable the trypanosomes to evade clearance by activated MNP. Consistent with this hypothesis, high levels of nitrite (a stable NO reaction product) were detected in the wells of the LPS-stimulated RAW264.7 cells, and this was reduced to background levels in the presence of indolepyruvate (Fig. 4B). Furthermore, treatment with indolepyruvate limited the ability of LPS-stimulated RAW264.7 cells to kill *T. brucei* (Fig. 4C). These data imply that ID LPS-treatment may have induced the production of pro-inflammatory mediators such as NO in local CSF1R + MNP, and these may have enhanced their ability to clear the parasites from the infection site^20^. However, it is plausible that the production of trypanosome-derived metabolites such as indolepyruvate may offer some protection against this.

## Discussion

Macrophages are considered to play an important role in protection during the early stages of African trypanosome infections in mammalian hosts by phagocytosing antibody-opsonized parasites, and producing cytotoxic and inflammatory mediators^21–23^. For example, the release of parasite-derived CpG DNA and soluble glycosylphosphatidyl inositol (GPI)-anchored variant surface glycoprotein stimulates the macrophages in a TLR-dependent manner to adopt a pro-inflammatory phenotype that is important for controlling the early stages of infection^21,24–26^. Although MNP are abundant in the skin, little is known of their impact on ID trypanosome infections^21^. We therefore studied the effects of increased MNP abundance or activation in the skin on susceptibility to ID trypanosome infection. We show that the CSF1-mediated increase in the abundance of MNP at the site of ID trypanosome infection did not affect the infection kinetics. However, bacterial LPS-stimulation in the dermis 24 h before ID trypanosome infection significantly reduced the parasitaemia, with only a proportion of the mice developing a detectable parasitaemia throughout the 30 d observation period. The stimulation of macrophages with bacterial LPS rapidly induces a pro-inflammatory transcriptional response^27,28^, and in vitro assays showed that LPS-stimulated RAW264.7 cells had enhanced cytotoxicity towards *T. brucei*. Together, our data suggest that the stimulation of dermal MNP with LPS may enhance their ability to eliminate ID injected *T. brucei* parasites in the skin.

Further studies are now required to identify the mechanism responsible for the LPS-mediated increased protection against ID trypanosome infection. Although only a small number of *Csf1r*-EGFP^+^ MNP were available for analysis, our data suggested that LPS stimulation did not affect the abundance of CSF1R + MNP in the dermis. Data from an ex vivo human skin organ culture model show that LPS-treatment induces the expression of high levels of pro-inflammatory cytokines, including interleukin (IL)-1β, IL-6, IL-8 and tumour necrosis factor-α^29^. Expression of these pro-inflammatory mediators may stimulate the activation of skin-resident innate immune cell populations, or aid the recruitment of neutrophils and other inflammatory MNP. Neutrophils, but not macrophages, express CD300f. highly in murine skin, and interactions between ceramide and CD300f. can suppress neutrophil accumulation and oedema in LPS-induced skin inflammation^30^. This implies that increased oedema and neutrophil accumulation are unlikely to be significant factors that contribute to the decreased susceptibility to ID trypanosome infection observed in LPS-treated mice. This is consistent with data suggesting that neutrophils do not contribute to the control of ID trypanosome infections^10^. Although neutrophils were rapidly recruited to the tsetse fly bite site after *T. brucei* infection^10,31^, these cells rarely engulfed the parasites and disease susceptibility was not affected by their absence^10^. Studies using mice with TLR-4 expression conditionally ablated in dermal MNP would help resolve whether the effects of LPS-treatment on ID *T. brucei* infection are specifically mediated through these cells.

Bacterial LPS is potent stimulator of the L-arginine NO pathway^32^, and in MNP this leads to the synthesis of high levels of the cytotoxic free radical NO via expression of the enzyme inducible NO synthase (iNOS)^33,34^. The production of high levels of NO by inflammatory MNP is important for the clearance of many intracellular pathogens. The macrophage response during the early stages of trypanosome infections in mice and humans is also accompanied by elevated NO production^35,36^. In vitro studies show that macrophage-derived NO can similarly mediate toxicity to extracellular trypansomes^37^. However, NO can bind to haemoglobin with high affinity, suggesting that in the extracellular environment of the mammalian host’s bloodstream, its toxicity against trypanosomes is reduced^15^. Macrophage-derived NO may also trigger iron loss within trypanosomes^37^, whereas another study has shown that it may react with serum albumin to produce S-nitroso-albumin that also has anti-parasitic effects^14^. Therefore, although NO may be inefficient at killing extracellular trypanosomes within the bloodstream, it could be highly effective within the microenvironment of the extravascular tissue spaces in the skin during the early stages following ID infection. In the current study, the enhanced ability of LPS-stimulated RAW264.7 cells to kill *T. brucei *in vitro also coincided with their ability to produce high levels of NO. Further in vivo studies, for example using iNOS-deficient mice or iNOS inhibitors, are clearly necessary to determine whether the effects of LPS-stimulation on susceptibility to ID trypanosome infection are mediated through enhanced NO production by MNP in the skin.

The duration of the effect of LPS-treatment on the MNP at the injection site was not determined. Soon after injection into the dermis by tsetse flies the trypanosomes can be visualised migrating towards and within the lymphatics in the skin^1^ and can be detected within the draining lymph nodes by 18 h^2^. In our study the mice were injected with a single dose of LPS into the dermis of the ear pinna. This would suggest that the effects of LPS stimulation were most likely restricted to the early stages of the infection within the skin dermis, and would have had little influence once the parasites had disseminated beyond this site.

While LPS-stimulated MNP may be effective in killing *T. brucei*, metabolites produced by the trypanosomes such as aromatic ketoacids could help to protect them against this activity^18,19^. For example, trypanosome-derived indolepyruvate inhibits the LPS-induced glycolytic shift in macrophages^18^. This leads to reduced expression of the transcription factor hypoxia-inducible factor-1α and decreased expression of pro-inflammatory mediators including NO^18,19^. Consistent with this potential immune evasion property, the treatment of LPS-stimulated RAW264.7 cells with 1 mM indolepyruvate reduced their ability to kill *T. brucei* and produce NO. However, since susceptibility to ID *T. brucei* infection was significantly reduced in the LPS-treated mice it is uncertain whether trypanosome-derived indolepyruvate had any influence during the early stages of the infection in the dermis. McGettrick and colleagues^18^ reported that the levels of aromatic ketoacids in the serum of *T. brucei* infected rats were typically in the range of 0.2–0.5 mM when their parasitaemias reached ~ 10^8^ parasites/mL. This would imply that the levels of indolepyruvate produced by the trypanosomes in the dermis soon after injection may be insufficient to have a significant impact on the responses of the local MNP. However, the concentrations and kinetics of such secreted molecules in extravascular microenvironments like the skin dermis would be an interesting aspect for further study.

Dermal CSF1-Fc treatment, in contrast, did not affect disease susceptibility despite increasing the abundance of MNP in the skin in the vicinity of the infection site. The stimulation of macrophages with CSF1 induces their polarisation towards an “alternatively-activated” or M2 phenotype, which includes the production of high levels of anti-inflammatory cytokines such as IL-10 and transforming growth factor-β^38,39^. These alternatively-activated macrophages can also dampen pro-inflammatory host responses towards certain pathogens^40^, and can secrete factors that mediate fibrosis or help repair the tissue damage caused by chronic infections with helminth parasites such as schistosomes^41,42^. In alternatively activated MNP, expression of the enzyme arginase competes with iNOS for the substrate arginine^43^, producing ornithine and urea instead of NO and citrulline. CSF1R-signalling in MNP similarly inhibits iNOS activity in MNP and skews the cells towards arginase expression^44^. A pro-inflammatory macrophage response is considered important for initial control during the early stages of a trypanosome infection^9^. Thus, it is plausible that CSF1-Fc treatment polarised the MNP in the dermis towards an alternatively-activated phenotype that may be less effective at clearing the ID-injected parasites from the skin. Despite this, disease susceptibility was not increased by CSF1-Fc treatment. As discussed above^1,2^, it is likely that there is only a short period between injection and the initial dissemination during which the MNP in the skin will have opportunity to significantly impact on disease pathogenesis.

Animal African trypanosome infections continue to inflict significant economic strain on sub-Saharan African livestock industries. Although a small number of trypanocidal drugs have been developed and are used in the field, drug-resistance can significantly impede their efficacy. We show that dermal LPS-stimulation significantly reduces susceptibility to ID *T. brucei* infection in mice. A thorough understanding of the cellular and molecular mechanisms that mediate this protection may lead to the development of novel strategies to help reduce the transmission of African trypanosomes.

## Materials and methods

### Mice

Six to eight weeks old female C57BL/6 J mice (Charles River, Harlow, England) and *Csf1r*-EGFP ‘mac green’ mice^12^ maintained on a C57BL/6 background were used where indicated. Mice were housed in individually ventilated cages with food and water provided ad libitum. All in vivo procedures were carried out under the authority of the appropriate project and personal licenses, in accordance with the United Kingdom Home Office regulations and the Animals (Scientific Procedures) Act 1986. Approvals for all the in vivo* studies* were obtained from The Roslin Institute’s and University of Edinburgh’s ethics committees, and these ensured they were carried out in compliance with the ARRIVE guidelines and recommendations.

### Trypanosomes

Pleomorphic wild-type *T. b. brucei* strain STIB247 were used throughout this study. These trypanosomes were originally isolated in 1971 from a hartebeeste (*Alcelaphus buselaphus*) in the Serengeti National Park, Tanzania^45^. The trypanosomes were axenically cultivated in vitro as previously described^1^. Prior to their use in each in vivo experiment, approximately 1 × 10^5^ axenically cultivated trypanosomes were first injected by the intraperitoneal route into C57BL/6J mice to obtain a fresh source of in vivo-adapted parasites. Blood was collected from these mice at the first peak of parasitaemia wave and used as a source of in vivo adapted trypanosomes in subsequent experiments. In these experiments, groups of female C57BL/6J mice were injected ID with approximately 1 × 10^5^ T*. b. brucei* STIB247 in vivo adapted parasites.

Blood parasitaemias were assessed at daily intervals using the rapid matching method^13^. Briefly, the number of trypanosomes was counted in wet blood films within 5 or 20 microscope fields depending on the parasite density. These values were then matched to the number of parasites/mL of blood based on data in an established reference table derived from haemocytometer counts of trypanosomes in infected blood samples. This assay has a minimum detection limit of approximately 4 × 10^5^ parasites/mL of blood.

### Intradermal injection with CSF1-Fc and bacterial LPS

Where indicated, female C57BL/6 J mice and *Csf1r*-EGFP^+^ mice (6–8 weeks old) were injected ID with 1 mg/kg of a recombinant porcine CSF1-Fc conjugate^46^ in a 10 µL volume into the ear pinna for three consecutive days. For bacterial LPS stimulation, mice were given a single ID injection into the ear pinna with LPS from *Escherichia coli* O111:B4 (10 µg/10 µL; Sigma-Aldrich, Gillingham, UK) in PBS.

### Whole-mount fluorescence multi-photon microscopy

Mouse ears were excised and immobilised on an imaging platform using tissue adhesive glue (3 M Vetbond, 3 M, St. Paul, MN, USA) and suspended in PBS before being imaged on a Zeiss LSM7MP 2-photon microscope (Carl Zeiss Ltd., Cambridge, UK). The microscope was equipped with a 20X/1.0NA water-immersion objective lens (Carl Zeiss Ltd), a coherent titanium-sapphire laser and an optical parametric oscillator (wavelength range 690 to 1400 nm). A laser output of 880 nm provided the excitation for the EGFP.

### RAW264.7 cells and co-culture with trypanosomes

Prior to use in studies, macrophage-like RAW264.7 cells^17^ were cultured in 100 mm^2^ plates (Sterilin Ltd., Newport, Gwent, UK) at 37 °C in a humidified 5% CO_2_/air atmosphere in 18 mL complete RPMI media (RPMI-1640, 5% heat-inactivated foetal bovine serum, 200 mM GlutaMAX, 5000 μg/mL penicillin–streptomycin; ThermoFisher Scientific, Paisley, UK). Cells were passaged every 3–4 days when they had reached a density of approximately 2 × 10^6^ cells/10 mL medium.

For co-culture experiments, RAW264.7 cells (1 × 10^5^) were stimulated with 10 µg LPS (or PBS as a control) and co-cultured with 1 × 10^6^ STIB247 trypanosomes per well in 24 well tissue culture plates (ThermoFisher Scientific). In some experiments indolepyruvate (indole-3-pyruvic acid; Sigma-Aldrich) dissolved in absolute ethanol was included at a final concentration of 1 mM. Triplicate wells were used for each treatment group. The plates were incubated for 24 h at 37 °C in a humidified 5% CO_2_/air atmosphere. The number of viable trypanosomes in each well was then counted using a haemocytometer. Experiments were repeated at least three times on separate days.

### Nitrite detection

Nitrite levels in culture supernatants were determined by Griess assay using a commercial kit (Griess Reagent System; Promega, Southampton, UK). Briefly, 50 µL of culture supernatant was added to triplicate wells of a clear 96-well plate. Plates also included a standard curve generated from serial dilutions of a nitrite standard ranging from 100–1.56 µM. Sulphanilamide solution (50 µL) was added to each well and incubated at room temperature for 10 min, before addition of N-1-napthylethylenediamine dihydrochloride solution. Plates were shaken and incubated for a further 10 min before absorbance was read at 550 nm.

### Statistics

Data are presented as mean ± SD. Unless indicated otherwise, statistical differences between groups were compared by Student’s t-test using GraphPad Prism v.8.0 software (GraphPad Software Inc. San Diego, USA). *P* values ≤ 0.05 were accepted as significant. A linear mixed effects model in RStudio (rstudio.com) was used to statistically compare the quadratic (squared) and cubic curve effect of the infected mouse parasitaemia across the observation period. Mean peak parasitaemias were compared by Student’s t-test.
